# Characterization of the Rate and Temperature Sensitivities of Bacterial Remineralization of Dissolved Organic Phosphorus Compounds by Natural Populations

**DOI:** 10.3389/fmicb.2012.00276

**Published:** 2012-08-10

**Authors:** Angelicque E. White, Katie S. Watkins-Brandt, Morgan A. Engle, Brian Burkhardt, Adina Paytan

**Affiliations:** ^1^College of Earth, Ocean, and Atmospheric Sciences, Oregon State UniversityCorvallis, OR, USA; ^2^Marine Science Program, Eckerd CollegeSt. Petersburg, FL, USA; ^3^Institute of Marine Sciences, University of California Santa CruzSanta Cruz, CA, USA

**Keywords:** heterotrophic bacteria, organic phosphorus, remineralization

## Abstract

Production, transformation, and degradation are the principal components of the cycling of dissolved organic matter (DOM) in marine systems. Heterotrophic Bacteria (and Archaea) play a large part in this cycling via enzymatic decomposition and intracellular transformations of organic material to inorganic carbon (C), nitrogen (N), and phosphorus (P). The rate and magnitude of inorganic nutrient regeneration from DOM is related to the elemental composition and lability of DOM substrates as well as the nutritional needs of the mediating organisms. While many previous efforts have focused on C and N cycling of DOM, less is known in regards to the controls of organic P utilization and remineralization by natural populations of bacteria. In order to constrain the relative time scales and degradation of select dissolved organic P (DOP) compounds we have conducted a series of experiments focused on (1) assessment of the short-term lability of a range of DOP compounds, (2) characterization of labile DOP remineralization rates, and (3) examination of temperature sensitivities of labile DOP remineralization for varying bacterial populations. Results reinforce previous findings of monoester and polyphosphate lability and the relative recalcitrance of a model phosphonate: 2-aminoethylphosphonate. High resolution time-series of P-monoester remineralization indicates decay constants on the order of 0.67–7.04 day^−1^ for bacterial populations isolated from coastal and open ocean surface waters. The variability of these rates is predictably related to incubation temperature and initial concentrations of heterotrophic bacteria. Additional controls on DOP hydrolysis included seasonal shifts in bacterial populations and the physiological state of bacteria at the initiation of DOP addition experiments. Composite results indicate that bacterial hydrolysis of P-monoesters exceeds bacterial P demand and thus DOP remineralization efficiency may control P availability to autotrophs.

## Introduction

The marine phosphorus (P) cycle is characterized by tight coupling between the uptake and decomposition of inorganic and organic P, respectively. Dissolved inorganic phosphorus (DIP), the end-product of heterotrophic remineralization of P-containing organic matter, is incorporated into a broad range of cellular compounds integral for energy storage, genetic material, and cell structure. Cell death and autolysis, exudation, viral lysis, and grazing all lead to the release of dissolved organic P (DOP) into the environment where it can be depolymerized, hydrolyzed, reassimilated, removed by absorption onto sinking particles, or it may accumulate in the surrounding environment. In this manner, the composition of the DOP pool in the ocean is largely controlled by the metabolic activity of microorganisms.

In the last decade, significant advances have been made toward furthering our understanding of the complexities of P-transformations in the sea. In a landmark work, Kolowith et al. (2001) used ^31^P nuclear magnetic resonance (NMR) spectrometry to show that the high molecular weight (HMW) fraction of dissolved organic matter (DOM) is dominated by P-linked esters (75%) and phosphonates (25%) occurring in unchanging proportions throughout the water column. Both components are known to have a biological origin: P-esters occur in both nucleic acids and membrane phospholipids while phosphonates are found as peptide, glycan, or lipid conjugates, typically in cell membranes (Hilderbrand, 1983; Quinn et al., 2007). In contrast, ^31^P NMR spectra of particulate organic material (POM) and laboratory cultures of marine phytoplankton indicate that P associated with living organisms is predominantly in the form of P-esters with relatively small to undetectable quantities of phosphonates (Benitez-Nelson, 2000; Paytan et al., 2003; Cade-Menun, 2005; Sannigrahi et al., 2006). The relative absence of phosphonates in POM and their abundance in HMW DOM coupled with the observation of the conserved proportionality of P-esters to phosphonates throughout the water column suggests that phosphonates are less biologically reactive and hence become selectively preserved in the DOM pool (Kolowith et al., 2001). This finding is also consistent with the “bottleneck” hypothesis of Karl and Björkman (2002), which proposes that the depolymerization of HMW DOP to potentially more labile, low molecular weight substrates largely regulates the turnover rate of the DOP pool in the ocean. Beyond these characterizations of the HMW DOM, methodological limitations have prohibited a full elucidation of marine organic P composition; rather, the classification of particulate and DOP (POP and DOP, respectively) currently relies on chemical reactivity, bond-structures, and/or relative abundance of compound classes (i.e., inorganic polyphosphate; Miyata and Hattori, 1986; Benitez-Nelson, 2000; Karl and Björkman, 2002). This uncertainty limits our ability to parameterize the turnover rate and flux of P in the marine environment as well as the relationship between organic matter decay and chemical composition.

Production and consumption can be tightly coupled such that some labile resources are undetectable in seawater (Søndergaard and Middelboe, 1995) or it can be uncoupled, to the extent that otherwise labile DOM can accumulate in seawater. Variability in rates and coupling of production and consumption of DOP results in both depth and spatial gradients in DOP concentrations. For example, the proportion of labile DOM, at least in terms of C, varies spatially with a gradient of decreasing concentrations from coastal to open ocean systems (Carlson, 2002). Strong seasonality in upper ocean DOP concentrations and community alkaline phosphatase activity (used as a proxy for labile DOP hydrolysis) has also been observed in the North Atlantic (e.g., Mather et al., 2008; Lomas et al., 2010; Sohm and Capone, 2010). Finally, in the North Pacific Subtropical Gyre (NPSG), rapid phosphate regeneration has been shown to follow exogenous additions of labile monophosphate esters (Björkman and Karl, 1994), which comprise ∼50% of the total dissolved P (TDP) pool in this region (Karl and Yanagi, 1997). This regeneration suggests strong uncoupling between DOP hydrolysis and P uptake by heterotrophic bacteria in the NPSG. So while the cycling and elemental composition of DOM (as carbon, nitrogen, or phosphorus rich substrates) is complex, these resources play an important role in surface biogeochemical cycles both as by-products of primary production and as fuel for heterotrophic bacteria. While efforts to elucidate the role of cycling of DOP in the ocean has illustrated the importance of this pool, much less is known about reactivity and decay rates of specific P-containing organic resources within the broader DOP “box” (Carlson, 2002; Karl and Björkman, 2002).

Generally, organic matter decomposition is thought to proceed in two stages: a rapid initial decomposition of labile pools followed by a slower decomposition of less reactive semi-labile organic matter. Labile dissolved organic pools turnover in a time span of hours to days, as they are depolymerized, consumed, or respired by heterotrophic bacteria (Bacteria and Archaea), and select eukaryotic phytoplankton and protists. However, the regeneration rates of specific compounds within the labile DOP pool and the environmental and biological parameters that may affect these rates are not well studied. To address this gap in our knowledge of P cycling, we have examined the short-term lability of a range of model DOP compounds; including P-linked esters and a phosphonate, as well as a short-chain inorganic polyphosphate. We have selected model P compounds that are known components of marine particulate and organic pools. A series of experiments were also conducted to estimate potential remineralization rates and temperature dependencies of labile DOP via natural heterotrophic populations isolated from both an oligotrophic (NPSG) and a coastal marine setting.

## Materials and Methods

### Field sample collection

Seawater containing heterotrophic populations was collected from within the euphotic zone (25 or 75 m) of the open ocean time-series Station ALOHA (A Long-Term Oligotrophic Habitat Assessment; 22°45′N, 158°00′W) site in the NPSG and from the upper 5 m at a hydrographic sampling station 10 miles offshore of Newport, Oregon (NH-10, 44°39′N, 124°17′W) in 2010 and 2011. For simplification all results referring to experiments conducted with populations collected from Station ALOHA offshore of Hawaii in the NPSG are referred to as NPSG, while those utilizing populations collected along the Newport Hydrographic line in the coastal waters offshore of Oregon are referred to as COASTAL. *In situ* temperature at the time of collection ranged from 22–24°C for Station ALOHA and ∼8–14°C for the Oregon coastal samples. Each respective sample was transferred from Niskin bottles (NPSG) or clean sampling buckets (Oregon) into 20 L acid-washed, autoclaved polycarbonate carboys, and transported to the laboratory at Oregon State University. All samples were kept under dark conditions at ∼24°C for a period of at least 1–2 weeks, until chlorophyll *a* fluorescence became undetectable. The holding period is also referred to as “aging.” Chlorophyll *a* concentrations were determined fluorometrically using a Turner Designs 10-AU fluorometer via the acidification method described by Strickland and Parsons (1972). The presence of heterotrophic bacteria after this initial dark incubation period was qualitatively spot-checked via enumeration of 4′,6-diamidino-2-phenylindole (DAPI) stained material collected onto a 0.2-μm black Nucleopore filter (Sherr et al., 2001). At time-zero and following the addition of organic substrates, samples were collected for quantitative flow cytometric analysis of bacterial concentrations following the methods of Sherr et al. (2001).

### Experimental design

Aliquots of select P substrates (targeted final concentrations = 1.0–5.0 μmol L^−1^) were added to seawater samples containing heterotrophic bacterial communities collected from Oregon coastal waters or the open ocean station ALOHA. Substrates added included a three-chain inorganic polyphosphate (P3, Pfaltz & Bauer S07555, ≥85%), two organic P-monoesters: d-glucose-6-phosphate (G6P, Sigma-Aldrich G7250, ≥98%), and adenosine-5′-monophosphate (AMP, Sigma-Aldrich A1752, ≥99%) as well as the phosphonate 2-aminoethylphosphonic acid (2-AEP, Sigma-Aldrich 268674, ≥99%). In all experiments, treatments and controls were incubated at a constant temperature of 24°C under dark conditions unless otherwise specified, for a period of 7–14 days to reduce the standing stock of photoautotrophs given that bottle enclosure and dark treatment are known to prevent growth of autotrophs (Smayda and Mitchell-Innes, 1974) and even lead to precipitous decline in picoplankton cell numbers (Calvo-Díaz et al., 2011). Additional details of the three successive experiments are as follows.

#### Experiment I

Initial experiments assessed the short-term lability of select P compounds introduced to coastal and oligotrophic populations of heterotrophic bacteria. Seawater was collected from the NPSG (25 m depth) and COASTAL (upper 5 m) sites in July of 2010 and experiments were completed within 3 weeks. Seawater temperature at the time of collection was 13.8°C for the COASTAL site and 22.5°C for the NPSG site. After 14 days, a total of 500 mL of COASTAL and NPSG seawater was amended with one of four P compounds (AMP, G6P, P3, or 2-AEP) to reach initial target concentrations of ∼5 μmol L^−1^. Duplicate incubations were carried out at 24°C in the dark and sampled over a period of 3 days. Changes in soluble reactive phosphorus (SRP), TDP, and bacterial cell numbers were measured. DOP was calculated as the difference of TDP and SRP.

Controls were conducted to examine abiotic hydrolysis, hydrolysis via free enzymes and changes in SRP concentrations in seawater with and without DOP amendments. NPSG and COASTAL seawater samples were autoclaved (120°C) for 30 min for abiotic controls and 5 μmol L^−1^ of AMP or G6P was added to 500 mL of seawater in duplicate. AMP and G6P additions were also made to 500 mL of <0.2 μm filtered seawater (not heat-treated) to evaluate potential hydrolysis in the absence of particles. SRP and TDP were tracked in controls at equivalent sampling intervals as experimental treatments.

#### Experiment II

Experiments were conducted with COASTAL seawater (more easily accessible via OSU) to parameterize remineralization kinetics of the more labile DOP compounds. The degradation of AMP and G6P to SRP at 24°C was monitored and the rate and timescale of remineralization was evaluated. COASTAL seawater (20 L) was collected on November 23rd 2010 and experiments were completed by December 7th 2010. The seawater temperature at the time of collection was 11.5°C. Seawater was stored at 24°C in the dark; after 7, 11, and 13 days, 500 mL volumes were subsampled into dark polycarbonate bottles and DOP was added at initial target concentrations of 5 μmol L^−1^ AMP, G6P, and AMP respectively. The concentration of SRP was then tracked at 10 min sampling intervals, using an automated valve to switch between treatments and controls (low P seawater with no added P). DOP and flow cytometry samples were collected at select time points throughout the experiment. The AMP additions were spaced a week apart in order to address the impact of aging on P monoester remineralization and the G6P (11 days) and AMP (13 days) additions were conducted as close as possible to compare remineralization rates between the compounds.

#### Experiment III

The temperature sensitivities of labile DOP remineralization by bacterial populations for coastal Oregon (AMP) and the NPSG (AMP and G6P) were examined. Seawater was collected from coastal Oregon (upper 5 m) and the NPSG (75 m) in November–December of 2011 and experiments were completed by January 2012. The seawater temperature at the time of collection was 23.1°C in the NPSG and 9.5°C at the COASTAL site. As in previous experiments, seawater was stored at 24°C in the dark for 14 days prior to the initiation of DOP additions. Sub-samples of 1 L of bacterial populations from coastal Oregon and Station ALOHA were acclimated at 9–10, 15, 20, 24, and 27–30°C for 2 days. Following this initial acclimation period, DOP additions (2 μmol L^−1^ of AMP for Oregon; 1 μmol L^−1^ of AMP; or G6P for Station ALOHA) were made to the 1-L bottles and split into 400 mL replicates for each treatment. The concentration of SRP was measured at 3 h intervals until SRP levels reached a plateau. Samples for DOP and flow cytometry were collected at select intervals.

### Analytical measurements

Soluble reactive phosphorus was measured as reduced phosphomolybdic acid in discrete samples according to the method of Strickland and Parsons (1972) using a Cary UV-VIS double beam spectrophotometer with a 1- or 10-cm pathlength as necessary. In order to capture rapid hydrolysis of highly labile DOP substrates, high resolution SRP concentrations were measured with a 5-cm pathlength Technicon AutoAnalyzer II™ in flow-thru mode (1.6 mL min^−1^) employing hydrazine as the reductant. The limit of detection (mean of a 3% NaCl blank + 3 × standard deviation of the blank; *n* = 12) for this system was 55 nmol L^−1^. An automated valve (Valco™) switched between controls (no exogenous P additions) and experimental treatments (+DOP) on 10 min sampling intervals.

Total dissolved P was determined by the alkaline persulfate oxidation method of Valderrama (1981). Dissolved samples were oxidized at 120°C for 40 min in Teflon^®^ digestion bombs, allowed to cool and run as discrete SRP samples as described above. The oxidizing agent used was a solution of potassium persulfate, sodium hydroxide, and boric acid added in a 1:10 oxidant to sample ratio. This method of TDP analysis has been shown to be sufficient in fully recovering simple monoesters as well as phosphonates (White et al., 2010). Non-reactive phosphorous (NRP) concentrations were calculated as the difference of TDP and SRP and are assumed to approximate DOP plus non-reactive inorganic P substrates (e.g., P3).

Bacterial abundance was determined by flow cytometry. Flow cytometry samples were collected in 4 mL cryovials, fixed with 60 μL of 10% paraformaldehyde and stored at −80°C until analysis. Briefly, samples were incubated with SYBR^®^ Green (10^−4^ dilution of a commercial stock) in the presence of 25 mmol L^−1^ (final concentration) of 0.2 μm filtered potassium citrate (Marie et al., 1997), vortexed, and allowed to sit in the dark for 15 min. The abundance of heterotrophic bacteria and high and low nucleic acid clusters [high nucleic acid (HNA) and LNA, respectively] were gated based on green fluorescence (515–545 nm) and side-scatter (both on a log scale) measured using a Becton–Dickinson FACSCaliber four-color flow cytometer with a 499-nm laser during a 3-min sample run at low flow rates (Sherr et al., 2001). Volume sampled was determined by the addition of yellow-green fluorescent microspheres (Fluoresbrite) of known concentrations (1–2 × 10^6^) and diameter (1.0 and 3.0 μm). True count polystyrene fluorescence standardization beads (6.0 μm) were used as a quantitation standard.

## Results

### Experiment I: Heterotrophic remineralization of select DOP compounds

Initial experiments compared the remineralization of select P compounds to controls with no added P in 24°C incubations. At time-zero, measured DOP concentrations (less the control, 0.10 ± 0.02 μmol L^−1^) in COASTAL incubations were 4.67 ± 0.26 μmol L^−1^ for AMP, 4.21 ± 0.16 μmol L^−1^ for G6P, 4.57 ± 0.02 μmol L^−1^ for 2-AEP, and 9.69 ± 0.39 μmol L^−1^ for P3 (due to a error in addition volume). Similarly for NPSG incubations, DOP levels at time-zero (less the control, 0.17 ± 0.11 μmol L^−1^) were 4.49 ± 0.16 μmol L^−1^ for AMP, 4.29 ±  0.36 μmol L^−1^ for G6P, 3.87 ± 0.40 μmol L^−1^ for 2-AEP, and 4.31 ± 0.16 μmol L^−1^ for P3. Over the full incubation period of 3 weeks, SRP concentrations in COASTAL and NPSG controls (no added P) were relatively low at 1.05 ± 0.3 and 0.22 ± 0.15 μmol L^−1^ respectively. Mass balance of P pools was achieved in all experiments (Table 1).

**Table 1:** **Experiment I data relevant to phosphorus mass balance: i.e., the net increase in SRP and net decomposition of DOP**.

	AMP	G6P	P3	2-AEP	Control
**NPSG EXPERIMENT I**					
Net SRP change, (μmol L^−1^)	4.19 ± 0.03	0.57 ± 0.03	1.40 ± 0.03	−0.07 ± 0.16	−0.03 ± 0.04
Net DOP change, (μmol L^−1^)	−4.24 ± 0.28	−0.66 ± 0.16	−1.69 ± 0.12	−0.87 ± 0.38	0.08 ± 0.04
% DOP degradation	91 ± 5%	13 ± 1%	31 ± 1%	NSD	NSD
*T*_0_ Bacteria mL^−1^ (%HNA)	7.8 × 10^5^ ± 2.0 × 10^4^ (18%)	8.5 × 10^5^ ± 2.4 × 10^4^ (19%)	6.3 × 10^5^ ± 7.1 × 10^4^ (18%)	6.8 × 10^5^ ± 6.5 × 10^3^ (18%)	7.1 × 10^5^ ± 6.3 × 10^3^ (13%)
*T*_F_ Bacteria mL^−1^ (%HNA)	2.6 × 10^6^ ± 4.8 × 10^5^ (50%)	1.1 × 10^6^ ± 1.4 × 10^4^ (42%)	1.0 × 10^6^ ± 2.6 × 10^4^ (36%)	1.1 × 10^5^ ± 2.0 × 10^4^ (39%)	1.1 × 10^5^ ± 2.2 × 10^4^ (36%)
Net increase of bacteria mL^−1^	1.9 × 10^6^ ± 3.4 × 10^5^	2.4 × 10^5^ ± 7.4 × 10^3^	4.1 × 10^5^ ± 4.7 × 10^4^	4.6 × 10^5^ ± 9.2 × 10^3^	4.1 × 10^5^ ± 8.7 × 10^3^
**COASTAL EXPERIMENT I**					
Net SRP change, (μmol L^−1^)	5.02 ± 0.19	3.27 ± 0.14	1.14 ± 0.16	0.28 ± 0.01	−0.02 ± 0.09
Net DOP change, (μmol L^−1^)	−3.80 ± 0.32	−3.99 ± 0.20	−0.99 ± 0.14	NSD	NSD
% DOP degradation	105 ± 4%	76 ± 3%	12 ± 2%	NSD	NSD
*T*_0_ Bacteria mL^−1^ (%HNA)	1.4 × 10^6^ ± 5.3 × 10^3^ (81%)	1.3 × 10^6^ ± 6.4 × 10^4^ (82%)	1.5 × 10^6^ ± 2.0 × 10^3^ (81%)	1.5 × 10^5^ ± 3.1 × 10^5^ (78%)	1.4 × 10^6^ ± 3.9 × 10^4^ (82%)
*T*_F_ Bacteria mL^−1^ (%HNA)	2.5 × 10^6^ ± 7.8 × 10^5^ (92%)	2.3 × 10^6^ ± 2.8 × 10^4^ (93%)	3.5 × 10^6^ ± 5.1 × 10^5^ (93%)	1.5 × 10^6^ ± 1.3 × 10^5^ (87%)	2.6 × 10^6^ ± 1.4 × 10^5^ (92%)
Net increase of bacteria mL^−1^	1.2 × 10^6^ ± 3.5 × 10^5^	9.2 × 10^5^ ± 4.6 × 10^4^	2.1 × 10^6^ ± 3.0 × 10^5^	1.5 × 10^4^ ± 3.4 × 10^3^	1.2 × 10^6^ ± 7.5 × 10^4^

**Figure 1:**
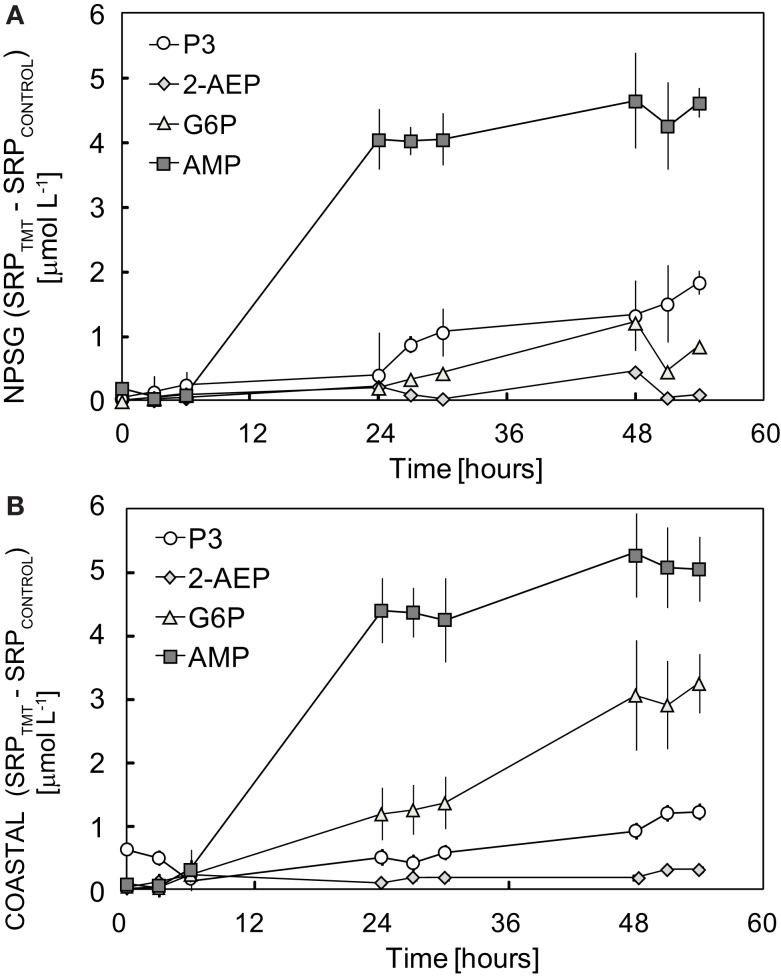
**The relative change (treatment-controls) in SRP levels after the addition of 5 μmol L^−1^ of 2-AEP, G6P, or AMP to duplicate 500-mL aliquots of seawater collected from (A) Station ALOHA in the NPSG or (B) COASTAL Oregon waters**. Initial additions of P3 were 5 μmol L^−1^ for NPSG experiment and 10 μmol L^−1^ for COASTAL experiments. Error bars represent the standard deviation of duplicate treatments.

*DOP was measured as the difference between TDP and SRP and includes non-reactive pools such as P3 (polyphosphate). Net differences are calculated as the mean of the duplicate SRP and DOP concentrations measured at the last three timepoints relative to the initial SRP and DOP for each treatment (see Figure 1). The percent of DOP degradation was calculated as the net change in SRP divided by initial DOP concentrations. Note that the initial P3 addition for the NPSG experiment was 5 μmol L ^−1^ whereas 10 μmol L^−1^ was added to COASTAL incubations. NSD indicates treatments where net P remineralization was not noticeably different than zero. Bacterial cell numbers and % high nucleic acid (HNA) cells are also presented for the initial ( *T*_o_) and mean of the final three timepoints ( *T*_F_). Standard deviations are calculated via error propagation techniques (Bevington and Robinson, 2003)*.

**Figure 2:**
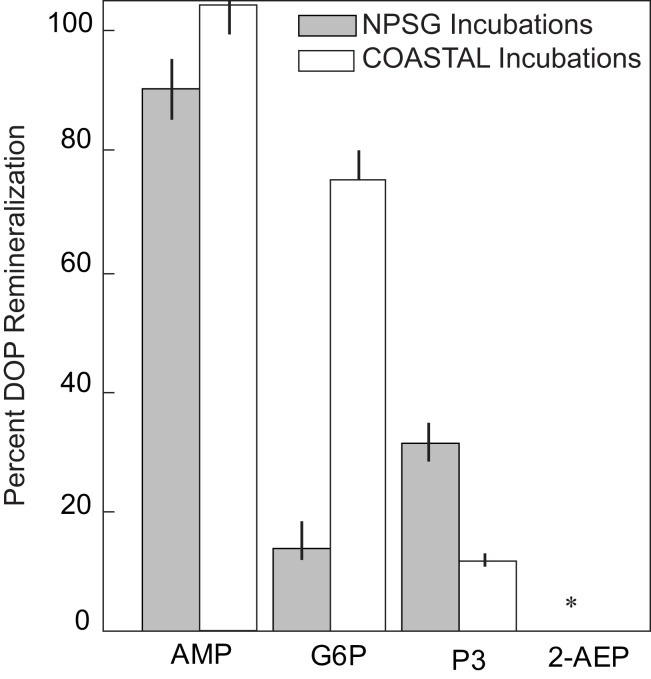
**The percentage of added DOP remineralized to SRP by populations of heterotrophic bacteria collected from the NPSG (gray bars) and COASTAL Oregon waters (white bars)**.The asterisk indicates that 2-AEP was not measurably remineralized by either microbial consortium. Error bars represent the standard deviation of duplicate treatments, calculating by error propagation of final SRP and added DOP measures for each replicate.

The difference in SRP levels between treatments and controls (SRP_TMT_–SRP_CONTROL_) over the course of each experiment is shown in Figure 1. AMP was rapidly remineralized in both NPSG and COASTAL experiments (91 ± 3% and 80 ± 5% decomposition of added DOP, respectively). Substantial remineralization of G6P (93 ± 5% decomposition of added DOP) was observed in COASTAL incubations and minimal turnover of P3 was seen in NPSG incubations (Table 1). 2-AEP was not appreciably remineralized by either NPSG or COASTAL populations (Table 1; Figure 1). Time lags between the addition of DOP and observation of remineralization in all cases was less than 24 h (Figure 1). Given that the aim of this experiment was a binary test of whether or not selected DOP compounds were hydrolysable, a rather coarse sampling resolution was adopted. As a result, the full exponential decay was not captured. Rather we have assessed the fraction of DOP that was remineralized by comparing the net change in SRP levels over the course of the experiment to initial DOP concentrations (Table 1; Figure 2). The relative utilization of AMP and G6P was significantly higher in COASTAL incubations relative to NPSG incubations (*t*-test *p* < 0.01). For P3, the relative utilization was higher in NPSG incubations, however given differences in initial P3 spikes, net SRP accumulation was similar (COASTAL = 1.14 ± 0.16 μmol P L^−1^; NPSG = 1.40 ± 0.03 μmol P L^−1^; Table 1). No appreciable P solubilization was observed in 2-AEP treatments (Table 1).

Initial bacterial concentrations were 7.3 × 10^5^ ± 8.5 × 10^4^ for NPSG incubations and 1.4 × 10^6^ ± 3.9 × 10^4^ for COASTAL incubations. Notably, initial bacterial concentrations in NPSG incubations are within the climatological range for Station ALOHA (3.1 × 10^5^–6.8 × 10^5^, data from 25 m horizon, 2006–2010 flow cytometry measurements)^1^. Similarly, initial bacterial concentrations in COASTAL incubations are within the range (1.7 × 10^6^–4.3 × 10^6^) reported for previous occupations of the NH-10 station (Longnecker et al., 2006; data from upper 6 m in September, 2006). In both NPSG and COASTAL incubations, increases in bacterial numbers were observed in both treatments and controls (Table 1). Bacterial numbers in AMP treatments were significantly higher than in other NPSG incubations (*t*-test, *p *< 0.005) and P3 treatments in COASTAL experiments showed substantial increases in bacterial numbers despite minimal remineralization. Nonetheless, the general pattern of change in bacterial numbers could not be correlated with relative DOP decomposition due to parallel increases in bacterial numbers in the control treatments (Table 2).

**Table 2:** **Experimental results from high resolution remineralization assays (Experiment II)**.

DOP Added (dark incubation period)	Heterotrophic bacteria – *T*_0_, Bacteria mL^−1^ (% HNA)	Heterotrophic bacteria – *T*_f_, Bacteria mL^−1^ (% HNA)	Net change of bacteria, Bacteria mL^−1^	DOP decay rate, day^−1^ (*T*_d_)	Remineralization time scale, Days
5 μmol L^−1^ AMP (7 days)	8.4 × 10^5^ ± 1.8 × 10^4^ (65%)	2.6 × 10^6^ ± 1.0 × 10^5^ (85%)	1.8 × 10^6^ ± 8.1 × 10^4^	7.04 ± 0.12 (0.14d)	0.41
5 μmol L^−1^ G6P (11 days)	7.4 × 10^5^ ± 2.4 × 10^4^ (57%)	2.4 × 10^6^ ± 5.2 × 10^4^ (92%)	1.7 × 10^6^ ± 8.3 × 10^4^	3.43 ± 0.13 (0.29d)	1.00
5 μmol L^−1^ AMP (13 days)	5.6 × 10^5^ ± 4.2 × 10^3^ (43%)	3.1 × 10^6^ ± 5.1 × 10^4^ (87%)	2.6 × 10^6^ ± 2.0 × 10^4^	4.47 ± 0.24 (0.22d)	1.06

*DOP decay rates, the duration of the dark incubation period between collection of water and DOP additions, turnover time (*T*_d_), and the remineralization time scale (*T*_0.1_ = the time required to remineralize ≥90% of added DOP) are presented. Also shown are the percent of total bacterial numbers that are HNA (high nucleic acid) at *T*_0_ and *T*_f,_ where *T*_0_ is the day that remineralization experiments are initiated, after aging of seawater and *T*_f_ corresponds to timepoints sampled after full remineralization of added DOP*.

**Figure 3:**
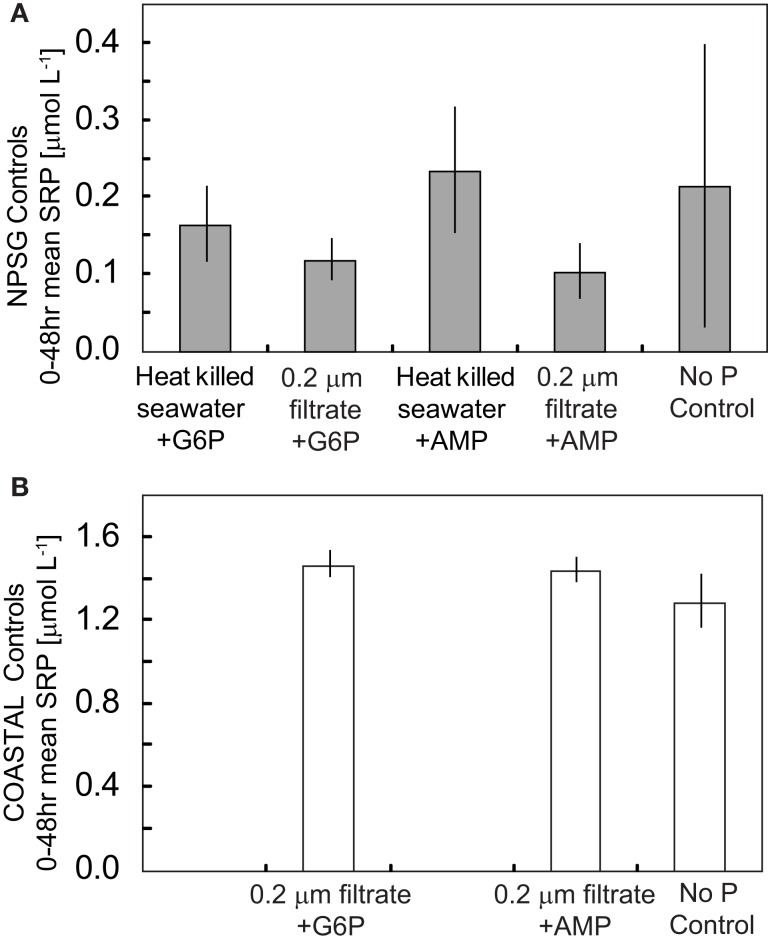
**Mean SRP levels over a ∼48 h incubation period in heat-killed controls and filtered controls relative to whole seawater samples to which no DOP had been added for (A) NPSG incubations and (B) COASTAL incubations**. No substantial remineralization of AMP or G6P was observed when natural populations had either been killed (autoclaved) or removed by filtration. Error bars represent the standard deviation of duplicate controls.

Given the rapid remineralization observed for P-monoesters, a series of controls were conducted to assess potential abiotic and free-enzyme (<0.2 μm filtrate) remineralization of AMP and G6P. Aliquots of AMP or G6P (5 μmol L^−1^) were added to heat-killed (120°C) NPSG and COASTAL seawater as well as to the 0.2-μm filtrate (not heat-treated) of these same seawater samples. In all cases the net change in SRP over a 48-h incubation period was not substantially different than the variability of SRP levels in unfiltered seawater with no added DOP (Figure 3). As a result, we conclude that the changes observed in Experiment I can be attributed to remineralization by active heterotrophic bacteria and Archaea populations rather than free-enzymatic activity or abiotic hydrolysis.

### Experiment II: High temporal resolution of AMP decomposition kinetics

The degradation of AMP and G6P to SRP in aged COASTAL water was monitored at high temporal resolution in order to estimate the range of labile DOP remineralization at 24°C. After 7 days, chlorophyll a concentrations were not detectable (data not shown), suggesting that dark incubation effectively selected against photoautotrophs. DOP additions of ∼5 μmol L^−1^ were made after 7, 11, and 13 days. Between 7 and 13 days, bacterial concentrations declined by 34 ± 2% whereas the percentage of bacteria classified as HNA content bacteria declined by 22% (Table 2).

**Figure 4:**
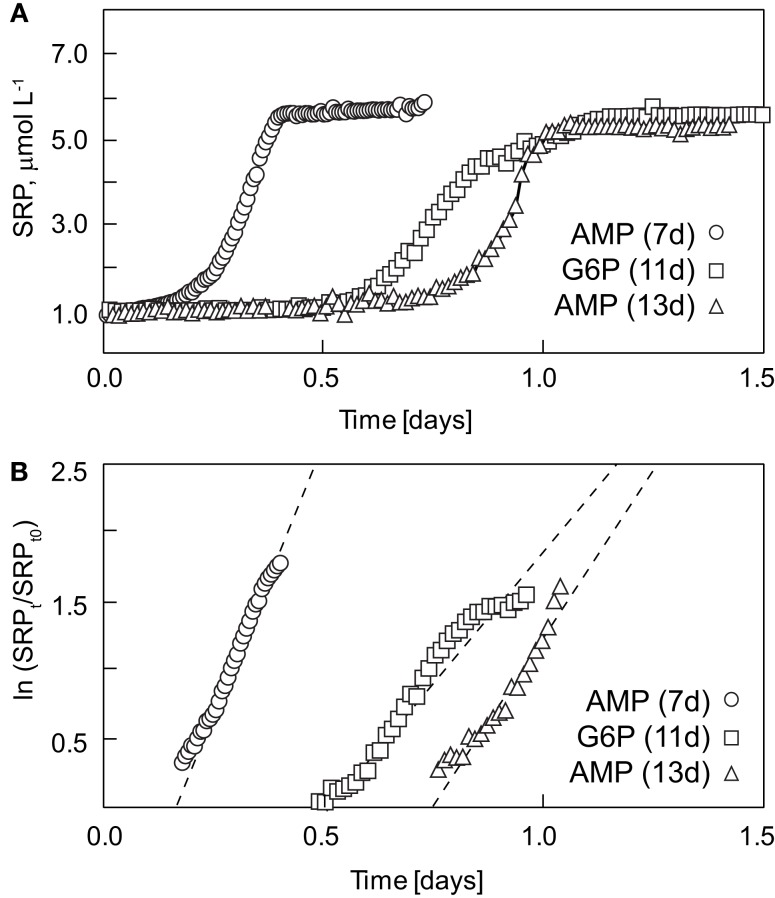
**High resolution time-series of AMP and G6P decomposition in COASTAL Oregon incubations**. Seawater was collected and stored at room temperature in the dark for a period of 7–13 days (values in parentheses in legend) prior to DOP addition. **(A)** The time-series of SRP remineralization are shown relative to **(B)** the natural log transform of the exponential phase of remineralization – e.g., the derivation of *k*_p_ constants.

We have calculated the remineralization time scale (*T*_0.1_), defined here as the time required to remineralize ≥90% of added DOP to SRP. We have compared AMP treatments initiated after 7 and 14 days to assess the impact of holding time or “aging.” As would be expected by reduced initial populations and potentially prolonged physiological stress after collection, the longer cells were maintained in the dark, the longer the time scale of remineralization (Table 2; Figure 4). *T*_0.1_ values shifted from 0.41 to 1.03 days as seawater was “aged” and initial populations decreased in number. The decay constant for DOP (*k*_P_) was derived by fit of the linear portion of natural log normalized SRP levels over time (Figure 4B). For AMP treatments, values of *k*_P_ decreased with increasing *T*_0.1_ and length of the incubation periods prior to DOP addition. Turnover time (*T*_d_) of AMP ranged from 3.4 to 7.2 h. We have also normalized the magnitude of SRP accumulation over the remineralization time scale to initial bacterial concentrations to account for cell-specific remineralization. The cell-specific P remineralization for AMP after 7 days was 13.6 ± 0.3 fmol P cell^−1^ day^−1^ whereas the rate after 13 days was 7.6 ± 0.1 fmol P cell^−1^ day^−1^ and so the decline in decay rates can not be fully explained by reduction in bacterial cells.

A second objective of this experiment was to compare remineralization rates of AMP and G6P which were aged for similar periods (11–13 days). Both AMP and G6P were fully remineralized within ∼1 day although SRP solubilization slowed at ∼0.85 day in the G6P treatment (Figure 4). The exponential decay constant for G6P (fit over the span of 0.5–1.0 day, *r*^2^ = 0.95) was 3.43 ± 0.13 day^−1^ whereas the decay constant for AMP-13 days (fit over the span of 0.75–1.05 d, *r*^2^ = 0.95) was 4.47 ±  0.24 day^−1^ (Table 2). Cell-specific G6P remineralization was 5.6 ± 0.2 fmol P cell^−1^ day^−1^, a rate significantly lower than that for AMP (7.5 ± 0.1 fmol P cell^−1^ day^−1^, *t*-test, *p* value <0.001).

While the initial standing stock of heterotrophic bacteria from this second sampling of COASTAL waters (winter 2010, 5.6 × 10^5^–8.2 × 10^5^) was lower than previous sampling (summer 2010, 1.8 × 10^6^), the increase in bacterial numbers over the incubation period was equivalent to or exceeded previous values (net change of bacteria mL^−1^ = 1.8 × 10^6^–2.5 × 10^6^, Table 2). Notably, we observed large relative increases in the percentage of HNA cells over the incubation period with HNA cells shifting from 43–67% of initial bacterial cell numbers to more than 85% by the end of each respective incubation period (Table 2).

### Experiment III: Temperature dependency of AMP and G6P decomposition

**Figure 5:**
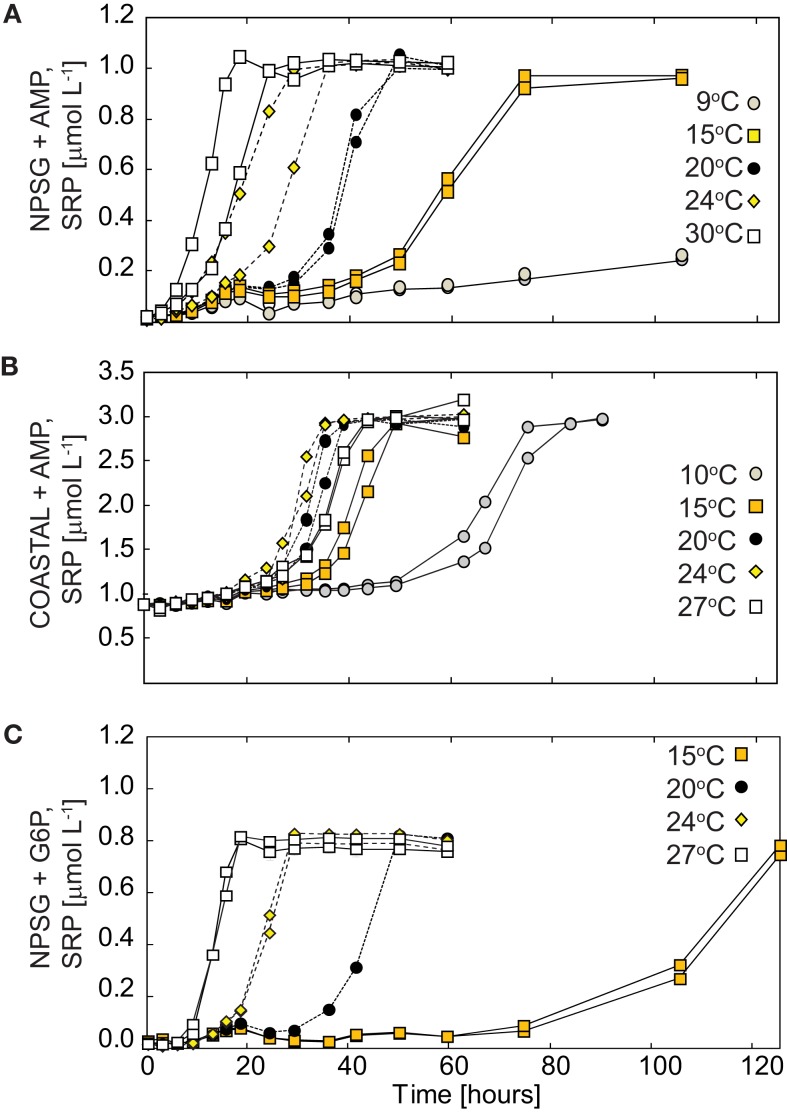
**Temperature dependent remineralization of AMP to SRP in incubations with seawater samples containing heterotrophic bacteria collected from the (A) NPSG and (B) COASTAL Oregon waters and (C) G6P remineralization with NPSG populations**. Approximately 1 μmol L^−1^ DOP was added to NPSG treatments and ∼2 μmol L^−1^ DOP to COASTAL treatments. Duplicate incubations were monitored for each temperature condition. Minimal changes in SRP levels were recorded after a 150-h incubation period in 4°C incubations using NPSG or COASTAL seawater (+AMP) and in 9°C incubations with G6P in NPSG seawater (data not shown).

The degradation of AMP to SRP was monitored at temperatures ranging from 4 to 30°C in incubations with heterotrophic bacteria collected from COASTAL and NPSG sites. G6P degradation was also assessed with NPSG samples. Initial DOP concentrations were 2.11 ± 0.06 μmol L^−1^ for AMP in COASTAL incubations and 1.04 ± 0.05 and 0.82 ± 0.01 μmol L^−1^ in NPSG AMP and G6P incubations, respectively. In both populations, P remineralization was minimal to undetectable in the 4°C treatment. Otherwise, with the exception of 9°C incubations of NPSG seawater, near-complete remineralization of AMP was observed at all temperatures (Figure 5, SRP accumulation/Added DOP = 96 ± 2% and 97 ± 3% in COASTAL and NPSG incubations, respectively). No substantive changes in SRP were observed in NPSG-G6P addition experiments at 4 or 9°C whereas in 15 to 30°C NPSG incubations 95 ± 4% of added G6P was remineralized (percent remineralization again calculated as net SRP accumulation/Added DOP; see Figure 5 for SRP accumulation). Remineralization time scales, the time required to decompose >90% of added AMP or G6P, are shown in Table 3.

**Table 3:** **Experimental results from temperature assays (Experiment III)**.

Incubation temperature °C	Heterotrophic bacteria – *T*_f_,	Turnover time,	DOP decay rate,	Remineralization time scale,
	Bacteria mL^-1^ (%HNA)	Days (*T*_d_)	Day^−1^ (*k*_p_)	Days
**COASTAL-AMP: *T*_0_ BACTERIA mL^-1^ = 3.6 × 10^5^ ± 6.0 × 10^4^ (46%)**				
4	NA	15.0	0.07 ± 0.01	NA
10	1.5 × 10^6^ ± 8.1 × 10^4^ (88%)	1.50	0.67 ± 0.05	3.2
15	1.1 × 10^6^ ± 3.6 × 10^5^ (81%)	0.74	1.34 ± 0.15	2.0
20	7.4 × 10^5^ ± 1.8 × 10^5^ (69%)	0.80	1.25 ± 0.06	1.5
24	3.0 × 10^5^ ± 8.1 × 10^4^ (48%)	0.80	1.24 ± 0.20	1.3
27	1.8 × 10^5^ ± 1.2 × 10^4^ (56%)	0.83	1.20 ± 0.15	1.6
**NPSG-AMP: *T*_0_ BACTERIA mL^-1^ = 4.5 × 10^5^ ± 7.2 × 10^4^ (39%)**				
4	NA	1.78	0.56 ± 0.15	NA
9	NA	1.52	0.66 ± 0.20	NA
15	1.1 × 10^6^ ± 8.6 × 10^4^ (80%)	0.87	1.14 ± 0.03	3.1
20	1.5 × 10^6^ ± 8.6 × 10^4^ (82%)	0.51	1.96 ± 0.02	2.1
24	1.1 × 10^6^ ± 6.9 × 10^4^ (77%)	0.26	3.86 ± 1.58	1.5
30	1.2 × 10^6^ ± 1.3 × 10^5^ (85%)	0.20	4.98 ± 1.23	0.8
**NPSG-G6P: *T*_0_ BACTERIA mL^-1^ = 4.6 × 10^5^ ± 6.2 × 10^4^ (40%)**				
4	NA	NA	0.13 ± 0.13	NA
9	NA	NA	0.14 ± 0.12	NA
15	1.1 × 10^6^ ± 6.0 × 10^4^ (78%)	1.24	0.81 ± 0.01	3.1
20	1.2 × 10^6^ ± 6.6 × 10^4^ (79%)	0.59	1.69 ± 0.22	2.1
24	1.6 × 10^6^ ± 9.1 × 10^4^ (83%)	0.26	3.89 ± 0.17	1.5
30	8.3 × 10^5^ ± 6.7 × 10^4^ (77%)	0.16	6.16 ± 0.09	0.8

*NA indicates treatments where remineralization was not complete; final bacterial samples were not collected for these treatments. Initial (T_0_) bacterial populations were measured in homogenous batches from NPSG and COASTAL waters prior to temperature acclimation and DOP additions and are presented in the heading for each treatment along with the fraction of bacterial counts clustering as HNA cells. Bacterial concentrations at T_f_ represent values after complete DOP remineralization*.

**Figure 6:**
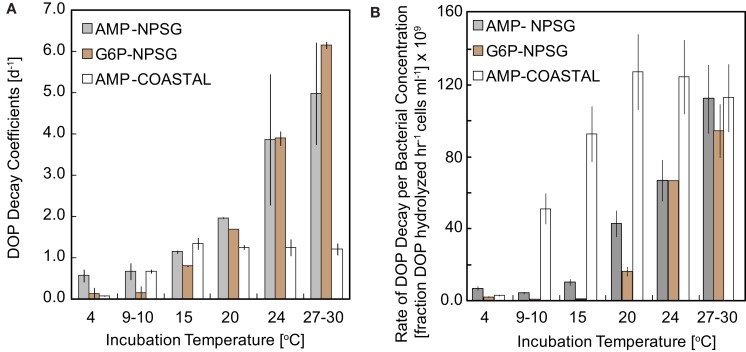
**(A)** Exponential phase AMP (NPSG and COASTAL) and G6P (NPSG only) decay coefficients derived for each temperature incubation (COASTAL populations were incubated at 4, 10, 15, 20, 24, and 27°C whereas NPSG populations were incubated at 4, 9, 15, 20, 24, and 30°C). Error bars represent the standard deviation of values derived from duplicate temperature treatments. The *in situ* temperature range in the upper euphotic zone at Station ALOHA is 19–28°C while surface temperatures at NH-10 in the coastal Oregon region range from 8 to 19°C. **(B)** The fraction of DOP remineralized per hour normalized to initial bacterial concentrations in each treatment. This calculation allows for normalization for bacterial concentrations and differences in DOP additions between NPSG (1 μmol P L^−1^) and COASTAL treatments (2 μmol P L^−1^).

The decay constants for AMP and G6P (*k*_P_) were derived by fit of the linear portion of natural log normalized SRP levels in replicates over time; standard deviations reflect deviation between duplicate incubations (Figure 6). Derived *k*_P_ values for AMP treatments were statistically similar in NPSG and COASTAL incubations at temperatures <20°C (*t*-test,*p* value = 0.83). COASTAL AMP turnover times (1/*k*_P_) ranged from 0.75 to 1.5 days at temperatures of 9 to 27°C with remineralization rates reaching a plateau at 15°C. The turnover of AMP in NPSG samples incubated at temperatures of 10 to 30°C ranged from 0.2 to 1.5 days, with no significant difference between the two higher temperatures assayed (24, 30°C, *t*-test *p* value = 0.39). These findings indicate that the NPSG populations are optimized for AMP depolymerization at ∼24°C whereas COASTAL populations operate more efficiently at ∼15°C. For the NPSG incubations, no remineralization of G6P was observed at 9°C whereas turnover time (0.2–1.2 days) at temperatures between 15 and 30°C was within the range measured for AMP (Table 3).

**Figure 7:**
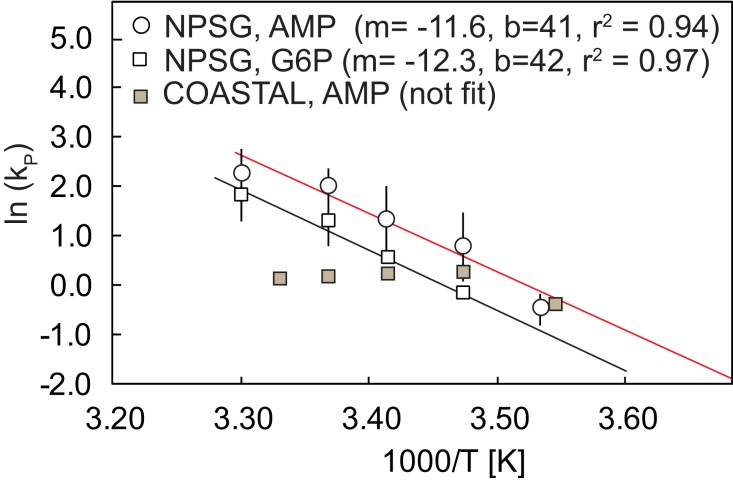
**Arrhenius plot of NPSG AMP (9–30°C) and G6P (15–30°C) and COASTAL AMP (9–27°C) decay rates determined at the sample’s original pH and salinity**. Error bars represent the standard deviation of values derived from duplicate temperature treatments. The slope (m), y-intercept (b), and *r*^2^ values for each linear regression are shown in the legend. COASTAL incubations were not regressed as decay rates were independent of temperature above 15°C, note however that at 9–15°C, temperature dependence for COASTAL AMP is similar to that observed for the NPSG AMP.

The remineralization time scale (*T*_0.1_) for NPSG incubations, the time required to decompose >90% of added AMP or G6P, varied linearly and inversely as a function of temperature (*r*^2^ = 0.92, *p* < 0.05), with a slope of −1.6 ± 0.2 h°C^−1^ (combining 9–30°C AMP and 15–30°C G6P treatments) such that the higher the temperature, the shorter the period required for DOP to be remineralized. In the case of COASTAL incubations, *T*_0.1_ values (1.3–1.6 days, Table 3) as well as *k*_P_ coefficients (1.2–1.25 day^−1^, Table 3) and cell normalized rates (Table 4) were independent of temperature at or above 15–20°C. An alternative means of presenting the temperature dependence of remineralization is via linear analysis of the Arrhenius plot of decay rates (*k*_P_; Figure 7). Fit of the natural log of *k*_p_ values to 1/*T* in units Kelvin results in the following equations for predicting AMP (at temperatures between 9 and ≤30°C) and G6P degradation (at temperatures between 15 and 30°C) by NPSG bacterial populations,

$$\begin{array}{rcl} \ln\left(k_{\text{p}}\right) & =-11630\;T^{-\text{1}}+\text{41}\;\text{ for AMP} & \text{(1)}\\ \ln\left(k_{\text{p}}\right) & =-12227\;T^{-\text{1}}+42\;\text{ for }G6P & \text{(2)} \end{array}$$

**Table 4:** **Cell-specific DOP remineralization and relative DOP utilization for Experiment I and III**.

Temperature (°C)	Days aged (Exp):	COASTAL, fmol P cell^−1^ day^−1^			NPSG, fmol P cell^−1^ day^−1^		
		DOP [μM]	AMP	G6P	DOP [μM]	AMP	G6P
4	14d (III)	2	0.14 ± 0.03		1	0.16 ± 0.03	0.05 ± 0.01
10	14d (III)	2	2.45 ± 0.41		1	0.10 ± 0.02	0.02 ± 0.00
15	14d (III)	2	4.45 ± 0.74		1	0.25 ± 0.04	0.02 ± 0.00
20	14d (III)	2	6.11 ± 1.02		1	1.03 ± 0.20	0.40 ± 0.06
24	7d (II)	5	13.55 ± 0.80				
24	11d (II)	5		5.19 ± 0.17			
24	13d (II)	5	7.49 ± 0.06				
24	14d (III)	2	5.98 ± 0.99		1	1.60 ± 0.27	1.60 ± 0.25
27	14d (III)	2	5.42 ± 0.91				
30	14d (III)				1	2.69 ± 0.46	2.27 ± 0.36

**Temperature (°C)**	**Days aged (Exp):**	**COASTAL, fraction of DOP hydrolyzed h^−1^ bacteria mL^−1^ × 10^9^**			**NPSG, fraction of DOP hydrolyzed h^−1^ bacteria mL^−1^ × 10^9^**		
		**DOP [μM]**	**AMP**	**G6P**	**DOP [μM]**	**AMP**	**G6P**

4	14d (III)	2	3.0 ± 0.5		1	6.9 ± 1.17	2.1 ± 0.3
10	14d (III)	2	51.2 ± 8.5		1	4.3 ± 0.7	0.8 ± 0.1
15	14d (III)	2	92.7 ± 15.5		1	10.5 ± 1.8	0.7 ± 0.1
20	14d (III)	2	127.4 ± 21.2		1	42.9 ± 7.3	16.3 ± 2.6
24	7d (II)	5	113.0 ± 2.4				
24	11d (II)	5		43.2 ± 1.4			
24	13d (II)	5	62.4 ± 0.5				
24	14d (III)	2	124.7 ± 20.8		1	66.9 ± 11.5	66.7 ± 10.4
27	14d (III)	2	113.0 ± 18.8				
30	14d (III)				1	112.4 ± 19.2	94.6 ± 14.8

*(Top) Rates of DOP remineralization normalized per cell for Experiment I and III relative to incubation temperature, the duration of aging, and the concentration of DOP added at the beginning of each incubation (**+**DOP). (Bottom) The fraction of added DOP hydrolyzed per hour per initial bacterial concentrations (adjusted by ×10^9^). This calculation accounts for differences in DOP stock additions and initial populations of heterotrophic bacteria*.

where *k*_p_ is the specific rate in units day^−1^ and *T* is in Kelvin. COASTAL populations were not regressed, however it is of note that the 9–15°C values fall along the NPSG AMP regression line (Figure 7).

As a final comparison, cell-specific hydrolysis was calculated in two ways (1) by normalizing the net change in SRP concentrations over the exponential phase to initial bacterial concentrations and (2) in order to account for differences in initial DOP levels, by normalizing the fraction of added DOP hydrolyzed per hour to initial bacterial concentrations (Table 4). The initial bacterial populations were lower and DOP additions were higher in COASTAL incubations as compared to NPSG incubations, and so net SRP remineralization per cell is elevated at all temperatures (COASTAL > NPSG, Table 4). When comparing the fraction of DOP hydrolyzed per hour by bacterial populations in these incubations (Table 4; Figure 6), relative AMP decomposition at temperatures 9–24°C still proceeded at higher rates in COASTAL populations; however NPSG and COASTAL incubations converge at 27–30°C (Table 4; Figure 6). Relative DOP utilization per bacterial concentrations reaches maximal values at 20°C for COASTAL populations and 30°C for NPSG populations.

## Discussion

Herein we have performed exogenous addition experiments to investigate the range of lability and remineralization of P compounds known to occur in particulate or dissolved fractions of marine organic matter: including the P-esters AMP and G6P (Kolowith et al., 2001; Sannigrahi et al., 2006), the polyphosphate P3 (Sannigrahi and Ingall, 2005; Diaz et al., 2008), and the phosphonate 2-AEP (Ternan et al., 1998; Quinn et al., 2007). The phosphonate 2-AEP has proven to be recalcitrant in both NPSG and COASTAL incubations suggesting that, at least on the time scales of days, this compound is relatively resistant to microbial degradation. This null finding may be a function of the enzymatic capacity of the microbial community captured in these incubations (e.g., lack of phosphatases in the sampled community) or chemical controls of gene expression or enzyme activity (e.g., inorganic P inhibition). Nonetheless, as 2-AEP degradation pathways have been described for both heterotrophic and autotrophic marine isolates (Ternan et al., 1998; Quinn et al., 2007; Singer et al., 2011) our finding of 2-AEP recalcitrance should warrant further study to constrain the broad phosphonate hydrolysis potential of marine heterotrophs. A simple first test would be to measure potential remineralization over longer timescales.

AMP, composed of a phosphate group, the sugar ribose, and a nucleotide, is known to be hydrolyzed by a class of enzymes broadly termed nucleotidases. In these experiments, in contrast to 2-AEP, we found that SRP was rapidly and consistently cleaved from AMP in incubations with microbial communities present in either NPSG or COASTAL seawater (Tables 1– 3). When normalized for initial DOP additions and bacterial concentrations, we find that the fraction of added DOP being utilized per hour by each population is similar at optimal temperatures (∼20°C for COASTAL and ∼30°C for NPSG; Table 4) although COASTAL populations decompose a larger fraction of added AMP per bacterial concentrations at lower temperatures (10–20°C) than NPSG populations. When comparing either decay constants or cell normalized rates, it is apparent that nucleotidase activity of COASTAL populations is adapted to a lower temperature range than NPSG populations (Tables 3 and 4; Figures 6 and 7). Assuming the cell-specific AMP rates reported here (Table 4), are realized in nature, it is also apparent that the activity of bacterial nucleotidases may supply the full complement of P needed to build a cell (12 fg P cell^−1^, Vrede et al., 2002) in a matter of hours. This finding is not new; the importance of bacterial nucleotidases as a means of P acquisition by marine bacteria is well described by Ammerman and Azam (1985, 1991); nucleotides are a documented component of marine DOP (Bjorkman and Karl, 2005) and even nmol L^−1^ additions of nucleotides have been shown to lead to significant P solubilization in freshly collected microbial communities (Björkman and Karl, 2003) suggesting that remineralization exceeds bacterial P demands. What our results add to the existing body of knowledge are (1) the finding that at least COASTAL nucleotidases do not appear to be saturated by micromolar additions of substrate: cell-specific decay rates increased at the same incubation temperature with increasing DOP additions (Table 4) and (2) nucleotidase enzyme activity is differentially regulated by temperature when mesotrophic and oligotrophic populations are compared (Figures 6 and 7).

Bacterial responses to G6P additions varied among treatments. Substantial remineralization of G6P was realized in experiments with COASTAL populations, however remineralization of G6P by NPSG populations was more variable (Figures 1 and 4; Tables 1– 4). Notably, initial SRP levels were not substantially different between collections (∼0.2 μmol L^−1^). When NPSG seawater was collected in summer (Exp. 1), 3.6 ± 0.13 μmol L^−1^ of residual DOP remained in the dissolved phase after the incubation period (initial DOP = 4.29 ± 0.75 μmol L^−1^) and the net change in SRP was modest (0.57 ± 0.02 μmol L^−1^, Table 1; Figure 1). However in subsequent experiments with microbial populations isolated in the winter from the NPSG, G6P was largely remineralized and SRP accumulated in the surrounding medium (Table 3). This is an intriguing finding that we cannot fully explain with the data in hand. There is precedent, however, in the literature for minimal P-ester remineralization by NPSG bacterial populations. Using a radiotracer approach applied to surface seawater collected from the NPSG, Björkman and Karl (1994) report negligible SRP accumulation in bacterially dominated size-fractions (<0.8 μm) following the addition of monoesters glycerophosphate, fructose-1,6-diphosphate, and G1P (glucose phosphorylated on the first carbon). Instead these authors found that SRP accumulation was most prominent in incubations with added nucleotides and/or P3 (the latter also observed herein, see Table 1). Variable hydrolysis of P-monoesters such as G6P may reflect different enzymatic capacities, differences in population structure, relative bacterial activities, and/or variable elemental status (P-replete or P-deficient) of initial microbial communities. As another parable for heterogeneity of enzymatic activity, Martinez et al. (1996) screened 44 marine bacterial isolates for protease, β-glucosidase, α-glucosidase, alkaline phosphatase (APA, a class of enzymes specific to P-monoesters such as G6P), lipase, and chitinase activity. Results with both free-living and particle-attached bacteria revealed an extremely broad range of cell-specific enzyme activities (e.g., 0.7–410 amol cell^−1^ h^−1^ for APA), leading the authors to conclude that shifts in bacterial community structure could strongly influence patterns of DOM hydrolysis in seawater (Martinez et al., 1996). Our results with G6P (e.g., seasonal differences in remineralization) are consistent with this sentiment, that is to say DOP hydrolysis likely varies widely between and among marine ecosystems due to changes in microbial communities which in turn are sensitive to chemical (nutritional sources, oxygen levels, etc.) and physical (temperature, turbulence, availability of particles) parameters.

We have sought to constrain some of the variability inherent to the rate of labile DOP hydrolysis via experimentation with different substrates and bacterial populations assayed over a range of incubation temperatures. Because we have added model substrates at potentially saturating concentrations (1–5 μmol L^−1^, at a minimum enhancement of 4× the standing stock of DOP) our approach yields only a potential rate of DOP hydrolysis – likely an upper constraint of *in situ* rates. We have also taken the approach of dark incubation to select against the activity of photoautotrophs. While the concentration of heterotrophic bacteria following these incubations is within the values observed for their collection sites and resultant rates of P remineralization are rapid and persistent, we have to caution that the cycling observed in a closed system may not precisely reflect the potential of the communities that were harvested; this is true for any incubation-based approach. This caveat stated, while derived G6P and AMP remineralization rates varied widely, the range was similar when the full data set for COASTAL (*k*_p_ = 0.67–7.04 day^−1^, *T*_d_ = 0.1–1.0 days) and NPSG incubations (*k*_p_ = 0.81–6.16 day^−1^, *T*_d_ = 0.1–0.9 days) are compared (Tables 1– 4). Normalizing per cell and for variability in DOP additions, we even find that populations converge to similar relative DOP degradation at optimal temperatures (Table 4).

Examining the results for the COASTAL incubations alone, some clear trends emerge: (1) remineralization rates and cell concentrations decline with prolonged stress of dark storage, however normalizing to initial concentrations of heterotrophic bacteria does not fully explain the depression in SRP remineralization rates (Table 4), (2) remineralization time scales generally decrease with increasing temperature – the warmer the water, the faster the decay of labile DOP, and (3) the exponential rate of AMP remineralization is temperature dependent between 4 and 15°C and temperature-independent above a 15°C threshold. Relative to this first point on dark incubations, it is possible that bacteriophage activity may have contributed to the decline in bacterial numbers and DOP remineralization rates as water was aged. While we did not sample for viruses, viral-induced mortality would reduce the standing stock of heterotrophic bacteria while concomitantly generating organic matter via cell lysis (“the viral shunt” pathway described in Wilhelm and Suttle, 1999). Preferential consumption of this “fresh” organic matter by uninfected bacteria would potentially reduce hydrolysis of added DOP. In addition to natural mortality and decline in cell-specific enzymatic rates, viral activity may help to explain the changes in potential cell-specific DOP hydrolysis in these experiments.

For NPSG incubations, major findings include the observation that (1) remineralization potential varies with shifts in initial bacterial populations, (2) the temperature threshold for measurable SRP remineralization is higher for G6P (15°C) than for AMP (9°C), and (3) when G6P (15–30°C) and AMP (9–30°C) remineralization is significant, the temperature dependence of both the ln(*k*_P_) and *T*_d_ can be described as a linear function of temperature (Eqs 1 and 2). Given the annual temperature range for COASTAL NH-10 (8–19°C, data from NOAA Station 46094)^2^, and for the euphotic zone (<125 m) in the NPSG sampling station ALOHA (19–28°C)^3^ temperature is most certainly a controlling factor of labile P remineralization. If model predictions of a warming ocean bear the scrutiny of time (Domingues et al., 2008), these findings may serve to parameterize the rate of P resupply to primary producers via heterotrophic decomposition.

The remineralization rates for labile DOP that we present here can be compared to previous *in situ* research and modeling studies. Hopkinson et al. (2002) conducted 180 days incubations of intact microbial communities isolated from coastal waters of the mid-Atlantic Bight and found that, on average, 32% of DOP was “very labile,” with “very labile” being defined as the fraction of DOP hydrolyzed at rates of 0.01–0.7 day^−1^. In accordance with a range of other studies (Clark et al., 1998; Paytan et al., 2003), Hopkinson et al. (2002) also demonstrate preferential remineralization of P relative to C and N. Over the course of 180-days incubations, 82% of the DOP pool was remineralized relative to turnover of 39% of the DON pool and 30% of the DOC pool. Preferential remineralization of P from DOP may partially explain why the surface ocean is rarely P limited. The rate of DOP turnover has also been estimated via numerical and isotope tracer methods. Using a data-assimilation approach, Luo et al. (2010) constrained the flux terms of heterotrophic microbial dynamics using measured DOM concentrations, bacterial biomass, and bacterial production for three ecosystems: the Arabian Sea, the NPSG, and the Equatorial Pacific. The turnover time of labile DOM (here in units of C) ranged from 1.0 to 1.2 days, with decay constants for labile DOM on the order of 0.21–0.36 day^−1^. While we have measured higher decay rates for COASTAL (up to 7.04 day^−1^) and NPSG populations (up to 4.98 day^−1^), the range of measured rates across varying communities, temperatures, and physiological conditions assayed herein are consistent with the findings of Hopkinson et al. (2002) and Luo et al. (2010). Our observation of elevated decay rates suggest that in nature, DOP remineralization may be limited by the composition of DOP compounds (e.g., substrate type and availability), sub-optimal temperatures for enzyme activity, competition with photoautotrophs, and/or the physiological state and density of the bacterial populations. Additional factors not directly studied here may include availability of inorganic nutrients and other non-P sources containing C and N.

To put these measured rates in further perspective, DOP concentrations in the surface waters in the NPSG are on the order of 200 nmol L^−1^ (Björkman et al., 2000), whereas DOP concentrations at the COASTAL NH-10 station were between 100–700 nmol L^−1^ over the course of the experiment. Given the observed range of remineralization rates (Tables 1– 4) and the liberal assumption that all measurable DOP is highly labile, the upper estimate for the residence time of labile DOP would be several hours. This is not unprecedented; DOP turnover rates obtained using cosmogenic isotopes show rapid recycling rates in surface waters (less than a day to 2 weeks; Benitez-Nelson and Buesseler, 1999; Benitez-Nelson and Karl, 2002). Similarly, isotope tracer and P uptake experiments also indicated DOP remineralization on the order or hours-days albeit with high spatial and temporal variability (Zohary and Roberts, 1998; Benitez-Nelson and Karl, 2002; Bjorkman and Karl, 2005; Sohm and Capone, 2010). The theme that emerges from these comparisons is that certain DOP compounds are rapidly decomposed into phosphate and residual organic matter. The rate of P remineralization appears to be largely in excess of bacterial P demand (as evidenced by inorganic P build-up) and as such would be available to fuel autotrophic growth. In the North Atlantic for example, where phosphate levels can be sparingly low (<10 nmol L^−1^), budgets of P fluxes and community alkaline phosphatase assays (heterotrophy + autotroph activity) indicate that DOP remineralization and/or direct uptake can support ∼25–30% of annual primary productivity (Mather et al., 2008; Lomas et al., 2010) and can be equivalent to the full complement of phosphorus export (Lomas et al., 2010). These studies and our own all point to heterotrophic remineralization of DOP as a significant control of P availability, oceanic productivity, and particle fluxes. Further efforts to elucidate the production and bioavailability of natural DOP pools across seasons and ecosystems will be necessary to more thoroughly constrain the controls of DOP decomposition in natural systems.

## Conflict of Interest Statement

The authors declare that the research was conducted in the absence of any commercial or financial relationships that could be construed as a potential conflict of interest.

## Footnotes

^1^http://hahana.soest.hawaii.edu/hot/hot-dogs/

^2^http://www.ndbc.noaa.gov/

^3^http://hahana.soest.hawaii.edu/hot/hot-dogs/
